# Vasopressor Therapy

**DOI:** 10.3390/jcm13237372

**Published:** 2024-12-03

**Authors:** Jean-Louis Vincent, Filippo Annoni

**Affiliations:** Department of Intensive Care, Erasme Hospital, Université libre de Bruxelles, 1070 Brussels, Belgium

**Keywords:** norepinephrine, vascular tone, arterial pressure, cardiac output, personalized, shock, vasopressin, angiotensin II

## Abstract

Vasopressor therapy represents a key part of intensive care patient management, used to increase and maintain vascular tone and thus adequate tissue perfusion in patients with shock. Norepinephrine is the preferred first-line agent because of its reliable vasoconstrictor effects, with minimal impact on heart rate, and its mild inotropic effects, helping to maintain cardiac output. Whichever vasopressor is used, its effects on blood flow must be considered and excessive vasoconstriction avoided. Other vasoactive agents include vasopressin, which may be considered in vasodilatory states, and angiotensin II, which may be beneficial in patients with high renin levels, although more data are required to confirm this. Dobutamine should be considered, along with continued fluid administration, to help maintain adequate tissue perfusion in patients with reduced oxygen delivery. In this narrative review, we consider the different vasopressor agents, focusing on the importance of tailoring therapy to the individual patient and their hemodynamic response.

## 1. Introduction

Vasopressors are widely used in critically ill patients. The only clinical indication for vasopressor use is to increase the vascular tone in the presence of severe hypotension associated with signs of altered tissue perfusion, manifested by clinical signs such as abnormal cutaneous perfusion, altered mentation, or decreased urine output, i.e., in shock. In these conditions, the lactate concentrations are increased, typically to greater than 2 mmol/L. The pathophysiological mechanisms of shock can be categorized into four groups with different hemodynamic alterations (Table 1). In the hypovolemic, cardiogenic, and obstructive types of shock, the cardiac output is typically too low to be able to maintain an adequate blood pressure, whereas, in distributive shock, the cardiac output is typically normal or high, and the vascular tone is reduced [1]. Importantly, patients with shock often have components of several underlying mechanisms.

The key equation when considering hemodynamic status and interactions is

arterial pressure = cardiac output × vascular tone.

It is important to remember this because the correction of hypotension should not result in a reduction in cardiac output and, therefore, in oxygen delivery (DO_2_) to the tissues.

## 2. Types of Vasopressors

### 2.1. Adrenergic Agents

**Figure 1:**
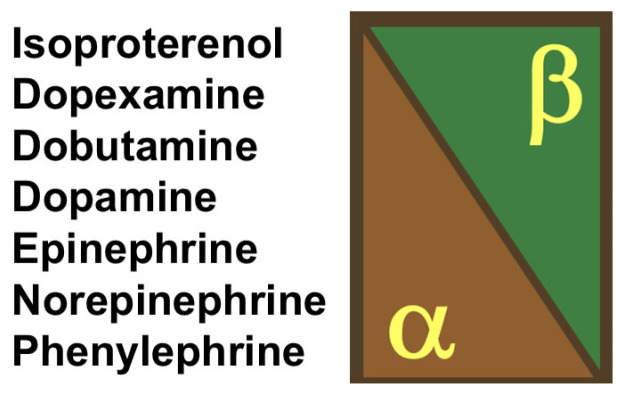
Schematic representation of the principal effects of adrenergic agents on the alpha- and beta-adrenergic receptors.

Adrenergic agents remain the first-line agents in severe hypotension as they have well-known, fairly reliable effects and a short half-life that facilitates their titration. The relative effects of the different agents on alpha- and beta-receptors are shown in Figure 1.

These differences in the stimulation of adrenoceptors provide the backbone to the current use of adrenergic agents. A highly selective alpha-agonist, such as phenylephrine, can provide potent vasoconstriction, but the increase in vascular resistance (afterload) can also decrease cardiac output. Such an event can be considered of small importance in settings in which the loss of vascular tone is transient (e.g., anesthesia induction in the operating room). However, it can rapidly become detrimental even during vasodilatory shock. Similarly, the selective beta-agonist isoproterenol reliably increases cardiac output but is associated with a loss of vascular tone.

Consequently, agents that have an effect on both receptors, with predominant but not exclusive alpha- or beta-activity, are often chosen as first-line agents. A summary of the most commonly used vasopressors and their corresponding infusion dose regimens is provided in Table 2.

Among the adrenergic agents, norepinephrine is generally preferred because of its additional mild inotropic properties (Table 3), which help to maintain cardiac output. A recent meta-analysis of 14 trials on the administration of norepinephrine in the treatment of septic shock [2] indicated that norepinephrine may be the preferred agent, with a minimal risk of arrhythmias.

Epinephrine, a powerful alpha- and beta-agonist, can be considered as an adjunctive or alternative vasopressor to norepinephrine, especially in situations where positive inotropism is required. It can indeed effectively increase arterial pressure and cardiac output. However, its administration can decrease the regional blood flow to the splanchnic and renal circulations, induce tachyarrhythmias, and increase cellular metabolism. Hence, the use of epinephrine is usually restricted to life-threatening hypotension, including the most severe form of acute circulatory failure, i.e., cardiac arrest. In a trial among 330 patients with septic shock randomized to receive either norepinephrine (with dobutamine when needed) or epinephrine, there was a (non-significant) trend towards an increase in mortality (40 vs. 34%) [3]. However, a strategy based on norepinephrine ± dobutamine enables the vasopressor requirements to be decoupled from the inotropic needs, which can be useful in certain patients and cannot be achieved with epinephrine alone. Nevertheless, epinephrine may represent a cost-effective alternative in complicated patients in units with limited resources [3].

Dopamine was a popular agent in the 1970s [4] because of its added dopaminergic effects that may theoretically improve kidney and splanchnic perfusion. However, dopamine did not fulfil its early promise; in particular, the protective effect on the kidneys has not been confirmed. In a large randomized multicenter trial in which dopamine was compared to norepinephrine as the first agent in circulatory shock, there were no benefits associated with dopamine use [5], and, in subsequent metanalyses in patients with septic shock, norepinephrine administration was associated with lower mortality rates than use of dopamine [6,7]. Some clinicians reserved dopamine for use in patients with relative bradycardia, but dobutamine is more commonly prescribed in this context. Hence, dopamine is no longer used.

Phenylephrine has very strong alpha-mediated vasopressor effects and is therefore highly effective in patients in whom there is a very abrupt development of severe hypotension, such as can occur during elective surgery. The increased vascular resistance associated with phenylephrine use may result in a decrease in cardiac output. The use of phenylephrine should therefore be limited to emergencies associated with profound acute hypotension.

### 2.2. Vasopressin

Vasopressin is not a “classical” vasopressor but a hormone that possesses vasopressor activity, as the name indicates. The administration of vasopressin has been proposed in the management of vasoplegic states, including septic shock. The pathophysiological basis for its administration is the observation that the blood vasopressin levels are within the normal range in septic shock but are very elevated in other forms of shock [8]. One hypothesis is that the neurohypophyseal stores of vasopressin become depleted in septic shock [9].

Because of its pharmacologic effects, vasopressin administration carries a risk of causing hepatosplanchnic ischemia, coronary syndrome, and cutaneous lesions associated with reductions in mesenteric, coronary, and skin blood flow, respectively. However, this effect may not represent a major problem when the cardiac output is high, as in septic shock. Indeed, vasopressin may have two major advantages over other vasopressors in this condition: one is a protective effect on the endothelium, resulting in less edema formation, and the other is renal protection [10]. Several studies have shown that urine output may increase during vasopressin administration. In a clinically relevant sheep model of septic shock due to peritonitis [11], we showed that vasopressin administration was associated with increased urine output and reduced fluid requirements, resulting in a less positive fluid balance, i.e., less edema, compared to norepinephrine or no vasopressor. Vasopressin was also associated with prolonged survival in this model. As expected, there was a reduction in mesenteric blood flow.

Some small studies have suggested an improvement in renal function with vasopressin administration [12]. In the randomized controlled VANISH trial [13], vasopressin administration was associated with a somewhat lower incidence of renal failure, including less need for renal replacement therapy (RRT), but the differences did not reach statistical significance. In a subsequent meta-analysis, Nagendran et al. [14] reported that vasopressin use was associated with a decreased need for RRT. This effect on the kidneys is attributed to a greater vasoconstrictive effect on the efferent than on the afferent arteriole, thus increasing the glomerular filtration rate [15]. Vasopressin also has the advantage of being associated with a lower risk of arrhythmias than adrenergic agents [14].

In a randomized controlled trial in patients with profound hypotension associated with vasoplegia following cardiac surgery, the administration of vasopressin significantly decreased the complication rates compared to the use of norepinephrine [16]. Many clinicians reserve vasopressin for use in patients with persistent signs of shock despite high doses of norepinephrine, but there is a sound rationale to use it early rather than later [17]. In the randomized controlled VASST trial [18], the use of vasopressin was associated with lower mortality than the use of placebo in less severe forms of septic shock (26.5% versus 35.7%) or when the lactate concentrations were not significantly increased [19] but not in more severe forms, suggesting that earlier use before shock worsens may be more beneficial. Other investigators have also reported a greater beneficial effect with early rather than late administration in studies using various databases [17,20].

Importantly, the dose of vasopressin should be limited to 0.03–0.05 units/min as higher doses could have harmful effects on the peripheral circulation. It has recently been suggested that the administration of a bolus of 1 unit may reveal the blood pressure response to a continuous infusion [21].

Terlipressin, a synthetic analogue of vasopressin, has been proposed because of its strong affinity for the V1a vascular receptors, thus being associated with fewer unwanted effects on other hormonal functions. Its long half-life can be an advantage in the management of the hepato-renal syndrome on the regular floor [22], but a molecule with a shorter half-life is preferred in the intensive care unit.

### 2.3. Angiotensin II

Angiotensin II (Ang II) is an old agent that was first proposed in the 1960s. Its very strong pressor effect, associated with the risk of excessive vasoconstriction, meant the molecule was not widely used until interest was reignited with the results of the ATHOS-3 trial in vasodilatory shock (primarily of septic [78%] or potentially septic [12%] origin) [23], showing its significant vasopressor effect in patients already receiving treatment with high doses of norepinephrine. The molecule was approved for use by the FDA and other licensing organizations on the basis of this vasopressor effect but without other clear indications for its use and no indication of any effect on other clinically relevant outcomes. The high cost of the drug (around EUR 1000 a day) means that some demonstration of superiority to adrenergic agents is necessary. It is clear that Ang II plays an important role in maintaining fluid and electrolyte homeostasis. It may also be associated with less myocardial oxygen consumption than norepinephrine [24]. However, it also has pro-inflammatory, profibrotic, and pro-apoptotic properties. Administration of Ang II may be part of a combined vasopressor strategy in which several vasopressors are used to accumulate the benefits of each agent and decrease the toxicity associated with high doses of any one molecule [25,26].

Interestingly, some studies suggested that Ang II administration may be particularly beneficial in patients with renal failure. In a post hoc analysis of the ATHOS-3 trial, in patients with renal failure requiring RRT, Ang II administration was associated with greater liberation from RRT and lower mortality [27]. This effect may be related to the increase in glomerular filtration pressure as a result of the preferential vasoconstriction of the efferent rather than the afferent arteriole, resulting in a net increase in glomerular filtration. In another pilot trial, in which patients receiving continuous RRT were excluded, 40 treated patients were compared with 80 matched controls to assess the effects of Ang II administration on plasma creatinine at day 7; there were no differences in the two groups [28]. Interestingly, in another post hoc analysis of the ATHOS 3 trial, in patients receiving high norepinephrine doses (i.e., >0.25 mcg/Kg/min), there was a beneficial renal effect (7-day discontinuation of RRT) in the Ang II-treated patients but not in the controls [29].

A further post hoc analysis of the ATHOS-3 study suggested a benefit in patients with acute respiratory distress syndrome (ARDS), with improved oxygenation in the Ang II-treated patients and a trend towards lower mortality [30]. Other studies have also suggested that Ang II administration may be associated with improved gas exchange [31]. Such an effect is biologically plausible in view of the high levels of angiotensin-converting enzyme (ACE) in the pulmonary endothelium, accounting for reduced Ang II levels in patients with acute respiratory failure [32]. It has also been suggested that Ang II potentiates hypoxic vasoconstriction, thus favoring a redistribution of intrapulmonary blood flow [33], compared to catecholamines.

The use of Ang II may be indicated particularly in patients with high renin levels. In another post hoc analysis of the ATHOS-3 trial, the use of Ang II was associated with a reduction in mortality compared to a placebo (50.9% versus 69.9%) in the 128 patients with renin levels above the median value [34]. Interestingly, increased renin concentrations after cardiac surgery were associated with hemodynamic instability and acute kidney injury [35], and, in another series of patients in whom vasopressors were started at anesthesia induction for cardiac surgery, Ang II reduced the circulating renin concentrations compared to norepinephrine, and high renin levels before surgery were associated with higher vasopressor requirements in the norepinephrine group but not in the Ang II group [36]. Further data are needed to explore the value of monitoring renin levels to select the most appropriate patients for this treatment and to evaluate the response to Ang II.

At present, the effects of Ang II in several categories of patients with vasoplegia have not been properly studied, such as patients with hemorrhagic shock, acute and acute-on-chronic liver failure, and patients with prothrombotic states, in whom the effects of Ang II remain controversial.

### 2.4. Nitric Oxide

Nitric oxide (NO) has a key role in the profound vasodilation associated with septic shock. Its effects are complex and not always deleterious. There are a number of possible methods of targeting the NO pathway. The non-specific blockade of NO synthase (NOS) with an investigational agent (546C88) resulted in the expected vasopressor effect but was associated with a significant increase in mortality [37], raising doubts about the place of such a strategy in these patients. Use of methylene blue (MB), an inhibitor of soluble guanylate cyclase, an enzyme involved in NO signaling, has also been tried in septic shock [38]. In a recent monocenter randomized trial in 91 patients with septic shock, MB administration was associated with reduced time to vasopressor discontinuation and more vasopressor-free days by day 28 [39]. However, in the absence of a clearly demonstrated beneficial effect, its use should be limited to randomized controlled trials [40]. It should be noted that MB-associated vasoconstriction can also have deleterious effects on the splanchnic territory that are correlated to the total dose, as suggested in a study using gastric tonometry in mechanically ventilated patients with septic shock [41].

Scavenging NO is another option. In patients with septic shock despite optimal fluid therapy, Simpkins et al. [42] recently reported vasopressor effects of a fluid comprising hydrophobic phospholipid nanoparticles (VBI-S), which reversibly absorbs NO. VBI-S administration was associated with an increase in MAP and enabled a reduction in vasopressor doses.

### 2.5. Other Vasopressors

Metaraminol, a sympathomimetic amine, is another strong vasopressor agent that was used many years ago and has now been virtually abandoned because of the same risks of excessive vasoconstriction. It is still used in some institutions, especially in Australasia.

Similarly, mephentermine, another sympathomimetic agent that acts by increasing norepinephrine release, was used in the past but is no longer widely available.

Hydroxocobalamin (vitamin B12) inhibits nitric-oxide-mediated vasodilation and has therefore been proposed for its vasoconstricting properties. However, the potential benefit of this approach is unproven [43].

## 3. Blood Pressure Targets

It is difficult to define a single blood pressure target that is optimal for all individuals. This has been well illustrated in clinical trials using different blood pressure targets in patients with septic shock. In the multicenter SEPSISPAM trial [44], patients with septic shock were randomized to a mean arterial pressure (MAP) resuscitation target of 80 to 85 mmHg (high-target group) or 65 to 70 mmHg (low-target group). As expected, there was no difference in outcome, presumably because some patients in the high-target group did not actually need such a high MAP and some patients in the low-target group did. A closer look at the MAP levels obtained in the two groups shows that the MAP was actually higher than the target (a common feature in such trials), i.e., around 85 mmHg in the high-target group and around 75 mmHg in the low-target group. Interestingly, in patients with a history of chronic arterial hypertension, a significant alteration in renal function, reflected by a doubling in plasma creatinine, occurred in 52.0% of the patients in the low-target group vs. only 38.9% in the high-target group. This observation suggests that a MAP even greater than 75 mmHg may be optimal in some patients, especially those with a history of hypertension. However, in another large trial in patients older than 65 years of age who were randomized to a target MAP of 60 to 65 mmHg (permissive hypotension) or usual care, there was again no significant difference in mortality [45], probably for the same reason as for the SEPSISPAM trial. Thus, from this trial, we can conclude that a MAP of even less than 65 mmHg may be acceptable in some patients, even when they are more than 65 years old. A recent meta-analysis of individual patient data confirms that a MAP of less than 65 mmHg may be tolerated and may even be beneficial in some individuals [46].

The inescapable interpretation of these results is that blood pressure targets should be individualized [47]. It is certainly acceptable to recommend an initial target MAP of 65 mmHg, but often the word ‘initial’ is forgotten and the subsequent individualized titration to more relevant and appropriate MAP values does not occur. In the 2017 Surviving Sepsis Campaign guidelines [48], we specifically wrote “we recommend an initial target MAP of 65 mmHg in patients with septic shock requiring vasopressors” and added “if initiated, vasopressor dosing should be titrated to an endpoint reflecting perfusion”. In our recent paper equilibrating the Surviving Sepsis Campaign guidelines with individualized care, we again stressed this point, writing that “although a mean value of 65 mmHg may be recommended as an initial goal, the optimal level may be higher in patients with a history of hypertension, atherosclerosis or chronic kidney disease. Conversely it may be lower in younger patients without previous vascular problems, in those with chronically low arterial pressure, or in whom adequate tissue perfusion is maintained” [49].

## 4. The Risk of Excessive Vasoconstriction

As noted in the Introduction, using vasopressor agents to target blood pressure without paying any attention to cardiac output and thus blood flow would be a profound mistake. This attitude may have led to the negative results of a prospective randomized controlled trial that evaluated the effects of selepressin in patients with septic shock [50]. This new vasopressin-related compound had shown very promising results in an earlier trial in patients with septic shock [51], but, unfortunately, the cardiac output was not assessed in the subsequent trial, and it is likely that some patients with a low cardiac output were harmed by excessive vasoconstriction. Any vasopressor agent may impair cellular oxygen delivery, so one should make sure that the cardiac output is kept close to, if not above, normal values.

**Figure 2:**
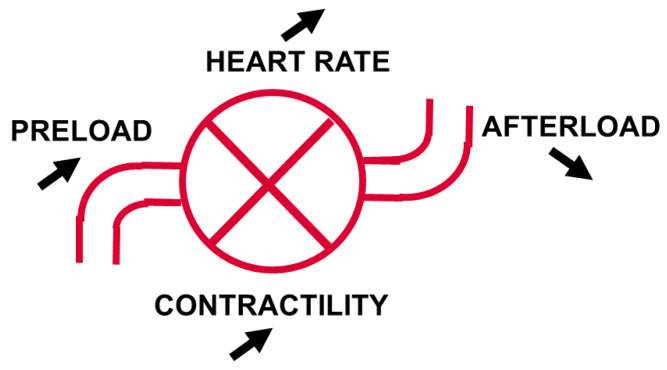
The four components of cardiac output and how it can be increased (arrows).

Considering the four determinants of cardiac output (preload, afterload, cardiac contractility, and heart rate) (Figure 2), if the doses of vasopressors cannot be decreased, increasing the heart rate is generally not a good option, and many of these patients already have tachycardia, so the two remaining options to improve cardiac output are to increase the preload and/or the contractility. For this purpose, the administration of intravenous fluids should be considered first, with a fluid challenge technique using small aliquots of about 200 mL of fluids [52]. If the filling pressures increase excessively, or if the tissue perfusion does not improve, the administration of an inotropic agent is necessary. A low central venous oxygen saturation (ScvO_2_) can represent a valuable tool to identify an inadequate DO_2_ [53]. Dobutamine is the most used and most reliable agent in this context, and low doses are usually sufficient. Interestingly, a recent meta-analysis suggested that the combination of norepinephrine plus dobutamine was associated with a lower risk of 28-day mortality than other vasoactive medications in patients with septic shock [7].

Other inotropic agents may be considered to increase tissue perfusion, including phosphodiesterase inhibitors (e.g., milrinone or enoximone) and levosimendan. Because of their longer half-life, associated with possible adverse effects on arterial pressure, despite their efficacy, these agents are used only cautiously.

## 5. Conclusions

The use of vasopressor support may be a lifesaving intervention in patients with shock, enabling an adequate tissue perfusion to be restored, but the effects of vasopressors on blood flow must always be considered. Focusing only on blood pressure is too simplistic. Moreover, the optimal arterial pressure varies from one patient to another and depends on other comorbid and current disease-related factors. Hence, vasopressor therapy should be individualized and closely monitored. Norepinephrine is the preferred first-choice agent, but it still has limitations. In vasodilatory states, the early use of vasopressin should be considered. The role of Ang II is still uncertain. Additional fluid administration and use of dobutamine should be considered to help maintain adequate blood flow to the tissues.

## Figures and Tables

**Table 1:** Problem-oriented first-line interventions (in addition to fluid challenges) for the four main categories of shock.

Principal Mechanism	Preferred Vasoactive Agent	(Some) Other Interventions
HYPOVOLEMIC	norepinephrine	source control (bleeding) transfusions (bleeding)
CARDIOGENIC	norepinephrine + dobutamine (consider PDE inhibitors, levosimendan)	coronary angiogram cardiac surgery arrhythmia control
OBSTRUCTIVE	norepinephrine	thrombolysis (pulmonary embolism) pericardial tap (tamponade) thoracic drain (tension pneumothorax)
DISTRIBUTIVE	norepinephrine (vasopressin?; angiotensin II?); add dobutamine in some cases	antibiotics source control

PDE = phosphodiesterase.

**Table 2:** Receptors and doses of most commonly used vasopressors.

Agent	Receptors	Commonly Used Dose
Norepinephrine	α (+++) and β (+)	0.1–5 µg/Kg/min
Epinephrine	α (+++) and β (+++)	1–60 µg/Kg/min
Dopamine	D1 and D2, β + α +	1–5 µg/Kg/min 5–15 µg/Kg/min >15 µg/Kg/min
Phenylephrine	α (+++)	0.1–1.5 µg/Kg/min
Vasopressin	AVPR (1a,1b,2)	0.01–0.05 U/min
Terlipressin	AVPR (1a>1b, 2)	20–160 µg/h
Angiotensin II	AT1R > AT2R	20–80 ng/Kg/min
Methylene blue	sGC	0.25–2 mg/Kg/h

AVPR: arginine vasopressin receptor type; AT1R and AT2R: angiotensin receptor types 1 and 2; D1 and 2: dopamine receptors 1 and 2; sGC: soluble guanylate cyclase.

**Table 3:** Presence or absence of inotropic effects among the most commonly used vasopressor agents.

No Inotropic Effect	Positive Inotropic Effect
Phenylephrine Metaraminol Mephentermine Vasopressin Angiotensin II Nitric oxide synthase inhibitors (e.g., LNMMA) Methylene blue	Norepinephrine Epinephrine Dopamine

## Data Availability

Not applicable.
